# Supplementary material for: A metamaterial-enabled design enhancing decades-old short backfire antenna technology for space applications
Source: Nat Commun. 2019 Jan 10;10:108. doi: 10.1038/s41467-018-08032-w (PMC6328586; doi:10.1038/s41467-018-08032-w)
Supplement: Supplementary file 1 — Supplementary Information [file 41467_2018_8032_MOESM1_ESM.pdf]

Supplementary Information

A metamaterial-enabled design enhancing decades-old short backfire antenna technology for space applications

Binion et al.

Supplementary Figures

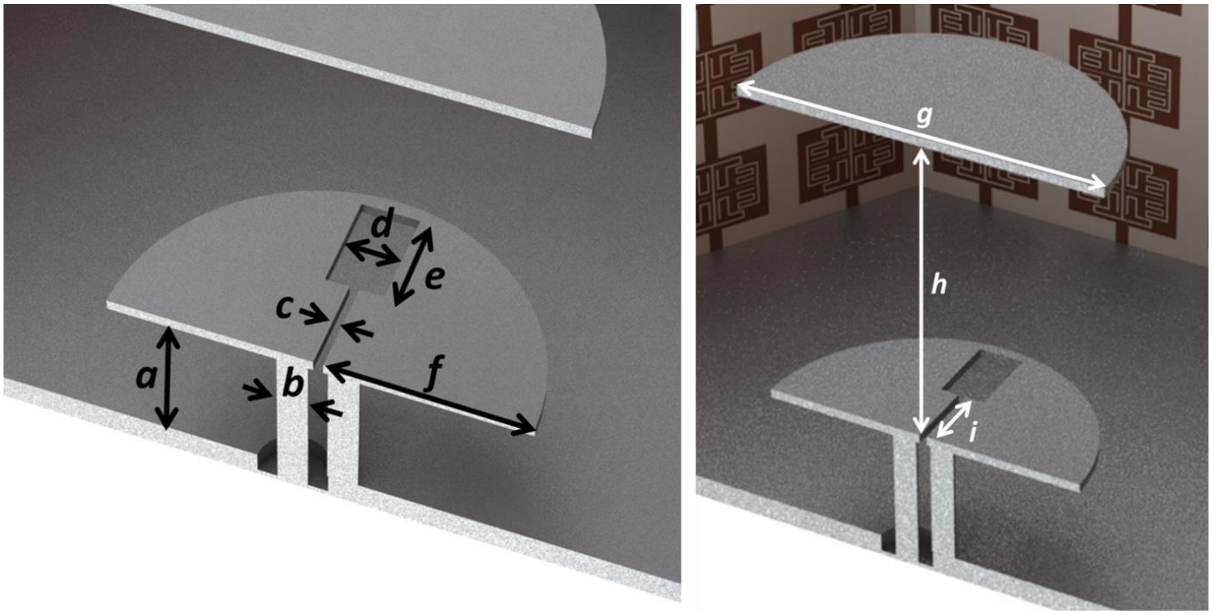

Supplementary Figure 1. Dimensions of A-SBFA feed antenna and subreflector.
Sizes of each dimension are listed in Supplementary Table 1.

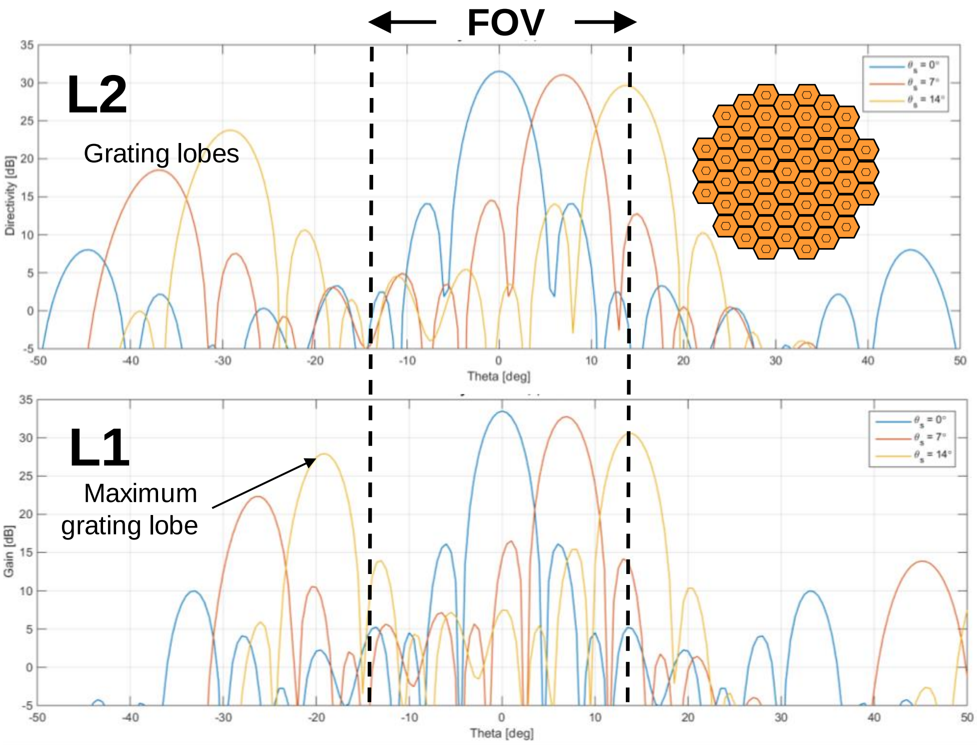

Supplementary Figure 2. Theoretical scanning performance of SBFA array with 2λ element spacing.

Medium Earth Orbit (MEO) satellite applications such as GPS require an antenna array with a field of view (FOV) of around +-14°. The above figure demonstrates that in the case of an array with 2λ element spacing, the grating lobes fall outside of the field of view when scanning to 14° radially at both L1 and L2.

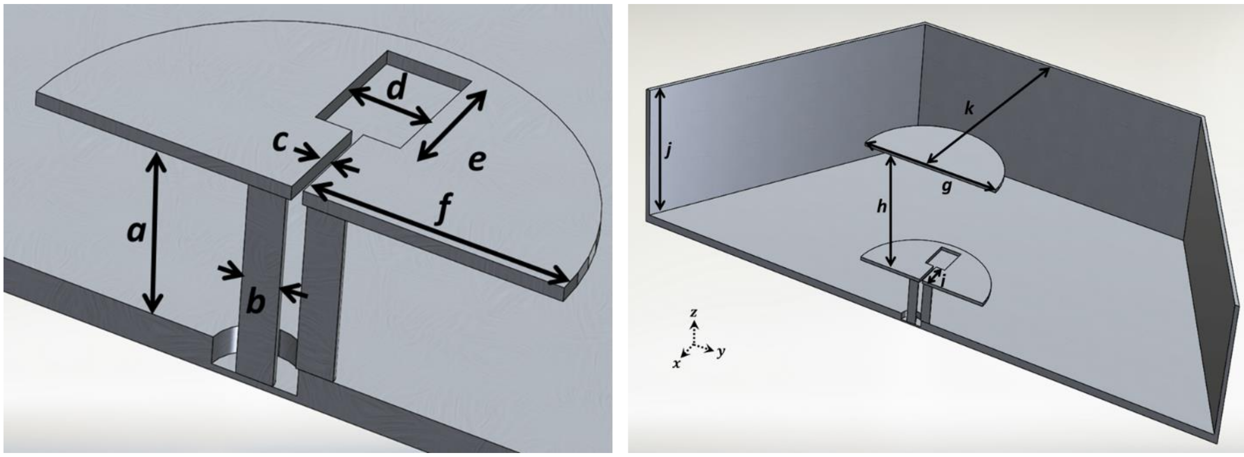

Supplementary Figure 3. Dimensions of SBF optimized with no metasurface.
Sizes of each dimension are listed in Supplementary Table 2.

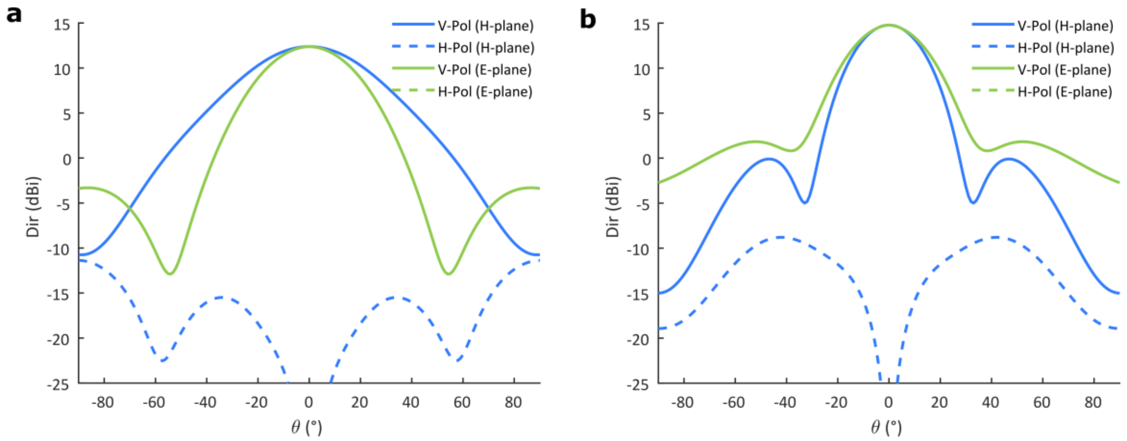

Supplementary Figure 4. Simulated directivity of optimized SBFA without metasurface liners. The dimensions of an SBFA without metasurface liners were optimized to maximize directivity at L1 and L2. A global optimization algorithm was run for several thousand iterations to achieve these results. Simulated directivity of this optimized antenna is shown here. The H-plane is the *x*-*z* plane in Supplementary Fig. 3. The E-plane is the *y*-*z* plane in Supplementary Fig. 3. **a.** Peak L2 directivity is 12.5 dBi, corresponding to an aperture efficiency of 67.2%. **b.** Peak L1 directivity is 14.8 dBi, corresponding to an aperture efficiency of 68.5%.

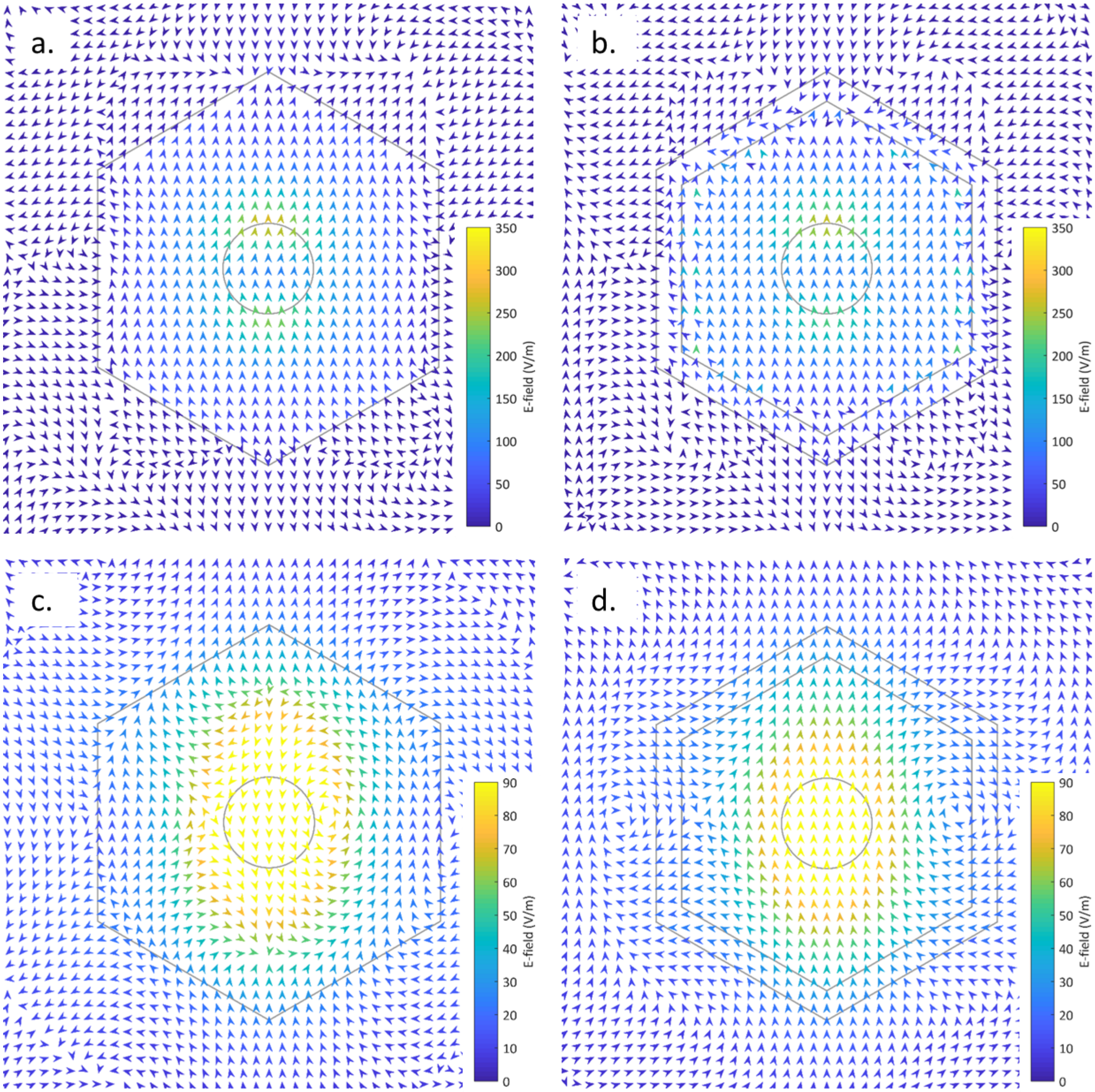

Supplementary Figure 5. Comparison of the aperture fields of the A-SBFA without and with the metasurface at frequency L2.

The vector electric fields for the hexagonal A-SBFA with and without the metasurface are shown. The metasurface helps to shape the aperture fields by enforcing a particular relationship between the incident electric and magnetic fields. This enables hybrid modes and allows for a more uniform aperture field distribution to be achieved. **a.** Shows the vector fields parallel to the *xy* plane 70 mm above the ground plane (between the patch and subreflector) for the A-SBFA without the metasurface. **b.** Vector fields parallel to the *xy* plane 70 mm above the ground plane for the A-SBFA with the metasurface. **c.** Vector fields parallel to the *xy* plane 240 mm above the ground plane (above the subreflector) for the A-SBFA without the metasurface. **d.** Vector fields parallel to the *xy* plane 240 mm above the ground plane for the A-SBFA with the metasurface.

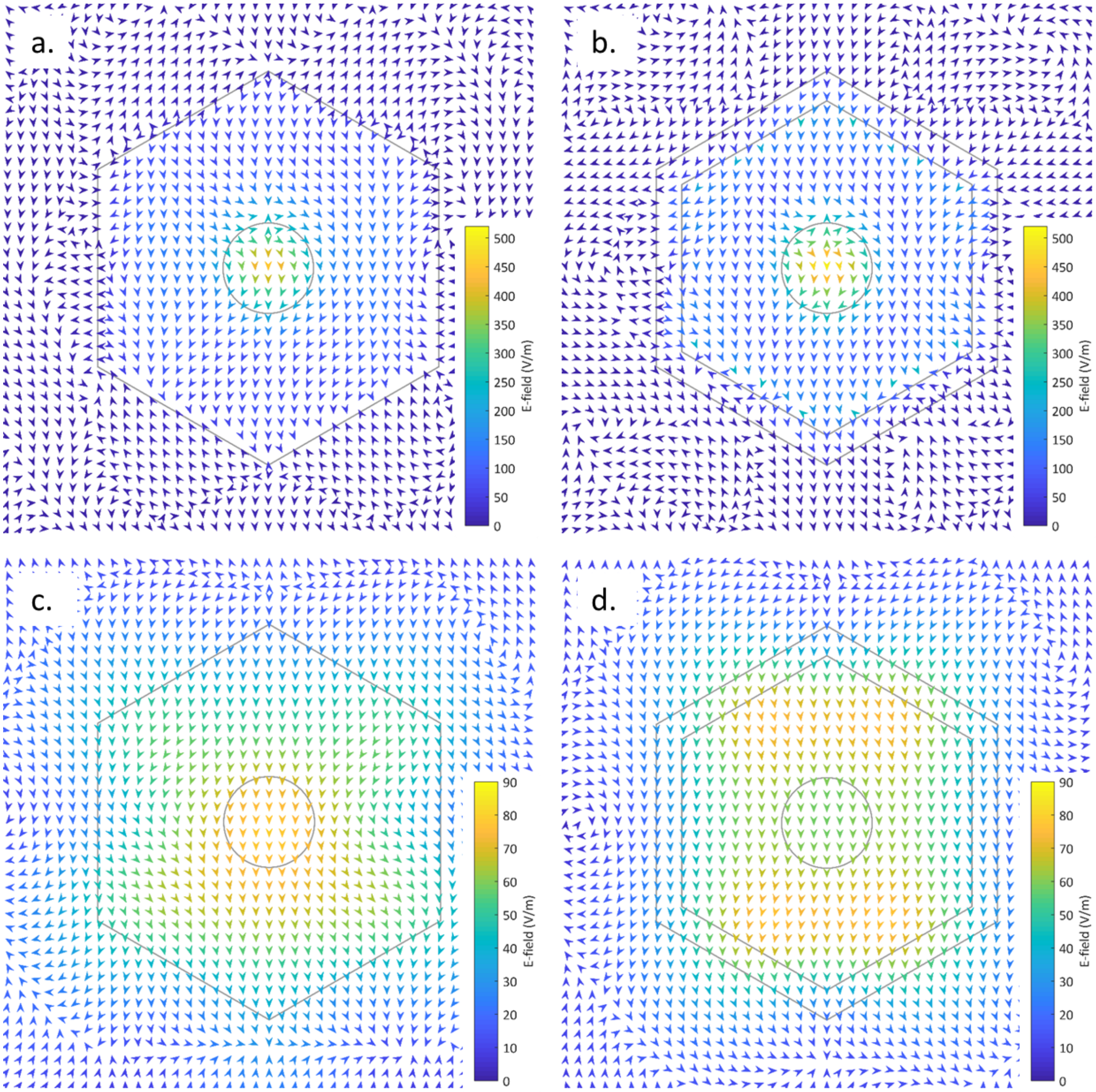

Supplementary Figure 6. Comparison of the aperture fields of the A-SBFA without and with the metasurface at frequency L1.

The vector electric fields for the hexagonal A-SBFA with and without the metasurface are shown. The metasurface helps to shape the aperture fields by enforcing a particular relationship between the incident electric and magnetic fields. This enables hybrid modes and allows for a more uniform aperture field distribution to be achieved. **a.** Shows the vector fields parallel to the *xy* plane 70 mm above the ground plane (between the patch and subreflector) for the A-SBFA without the metasurface. **b.** Vector fields parallel to the *xy* plane 70 mm above the ground plane for the A-SBFA with the metasurface. **c.** Vector fields parallel to the *xy* plane 240 mm above the ground plane (above the subreflector) for the A-SBFA without the metasurface. **d.** Vector fields parallel to the *xy* plane 240 mm above the ground plane for the A-SBFA with the metasurface.

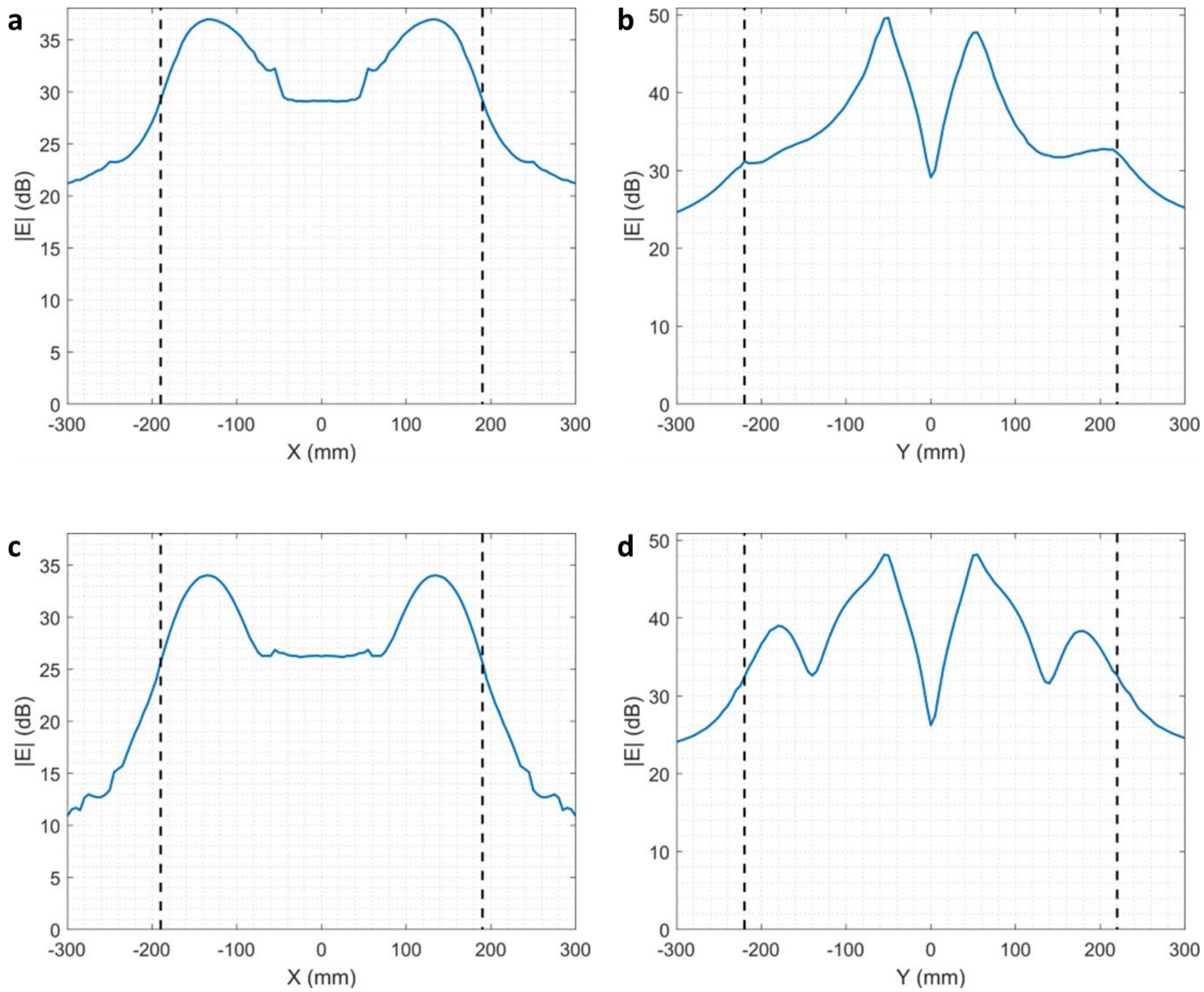

Supplementary Figure 7. Electric field magnitude cuts at L1 and L2.
The above plots show the magnitude of the electric fields 8 mm above the subreflector given an input power of 1 W. Dashed lines indicate the edge of the SBF aperture. **a.** Electric field magnitude at L2 in the H-plane. **b.** Electric field magnitude at L2 in the E-plane. **c.** Electric field magnitude at L1 in the H-plane. **d.** Electric field magnitude at L1 in the E-plane.

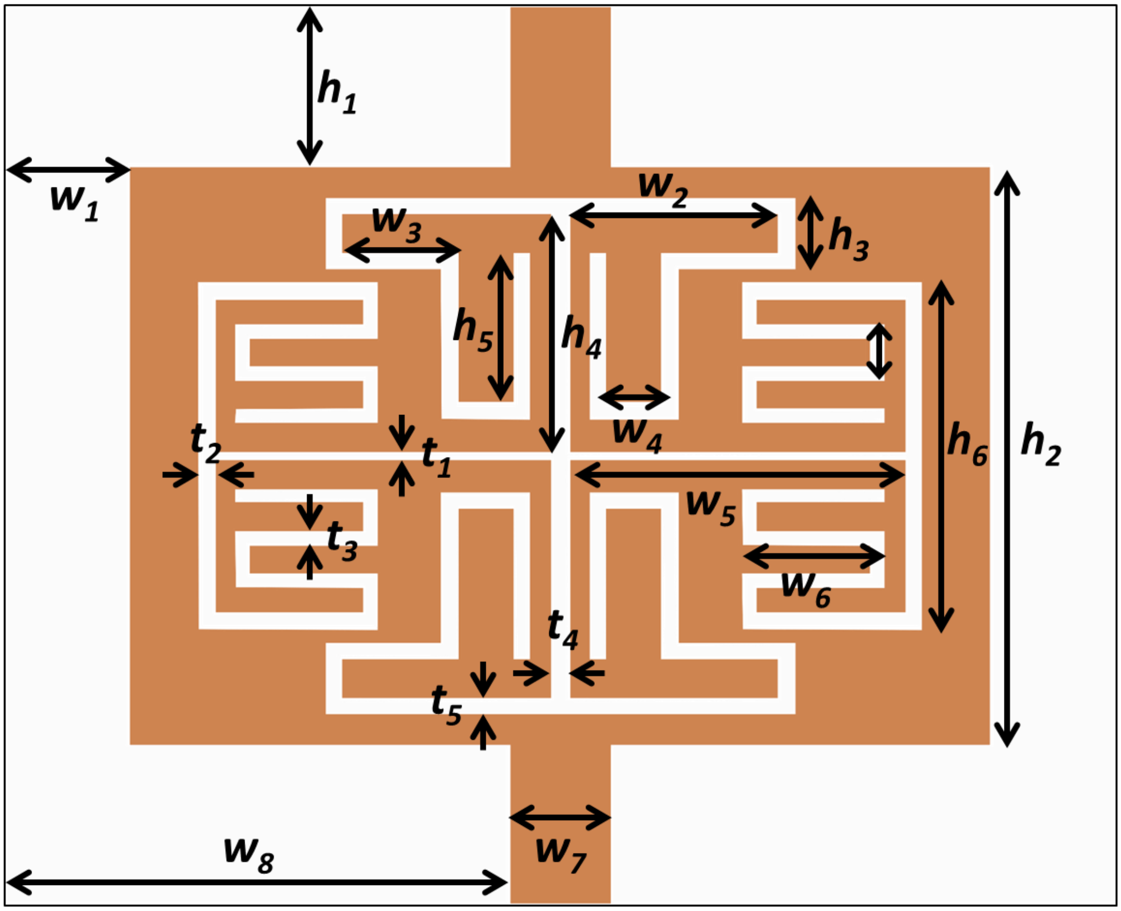

Supplementary Figure 8. Dimensions of metasurface unit cell.
Sizes of each dimension are listed in Supplementary Table 3.

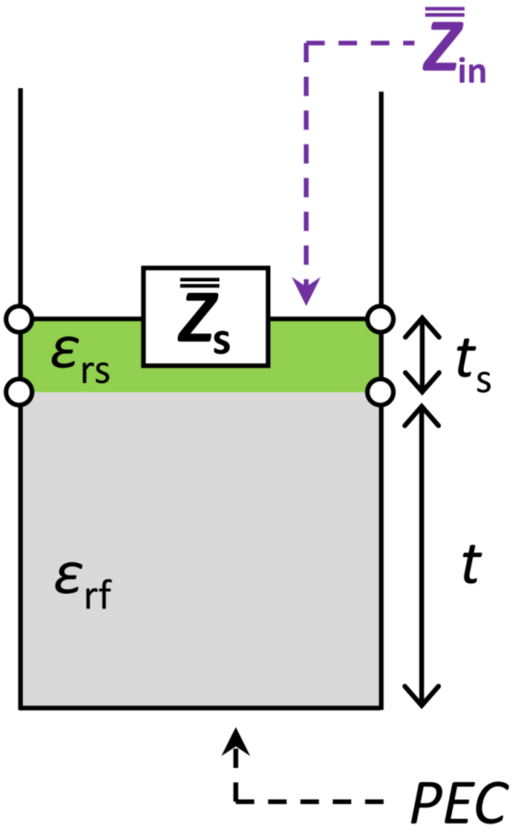

Supplementary Figure 9. Equivalent transmission line circuit used to extract surface impedance of metasurface.

The metasurface in an infinitely periodic environment can be represented by an equivalent transmission line circuit, in order to calculate the surface impedance tensor, Z_s, from the reflection coefficient of the system. In this design, t_s = 0.1676 mm, t_f = 33.6 mm, ϵ_{rs} = 3.66, and ϵ_{rf} = 1.04.

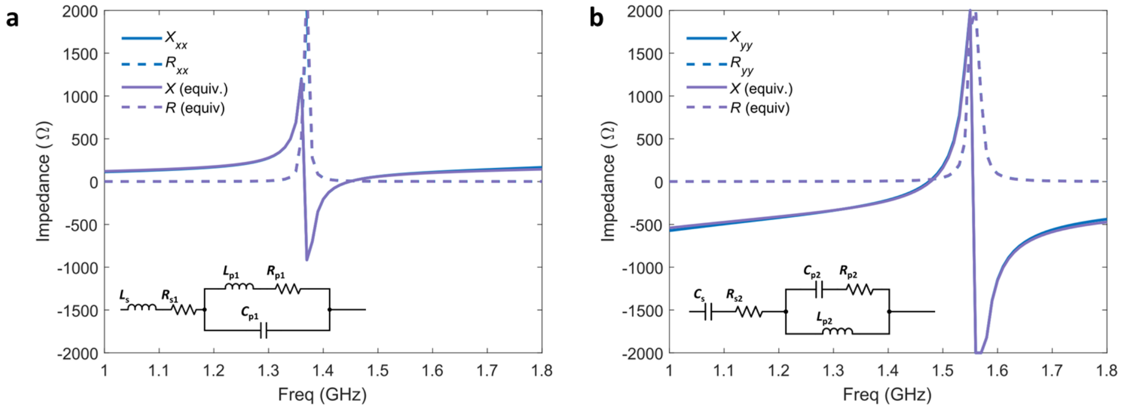

Supplementary Figure 10. Comparison of the extracted surface impedance to the impedance of an equivalent circuit.

a. Because the unit cells are electrically connected in the *x*-direction, the surface impedance can be modeled as an inductor in series with a parallel *LC* circuit that has a small resistive component. In the diagram above, L_s=15.25 nH, R_{s1}=1 Ω, L_{p1}=1.83 nH, C_{p1}=7.38 pF, R_{p1}=0.09 Ω. **b.** Similarly, since there is a gap between unit cells in the *y*-direction, the *y*-directed surface impedance can be modeled as a capacitor in series with a parallel *LC* circuit that has a small resistive component. In the diagram above C_s=0.271 pF, R_{s2}=0.1 Ω, L_{p2}=4.32 nH, C_{p2}=2.41 pF, R_{p2}=0.27 Ω. The extracted surface impedance agrees well with the impedance values calculated using the equivalent circuit models shown.

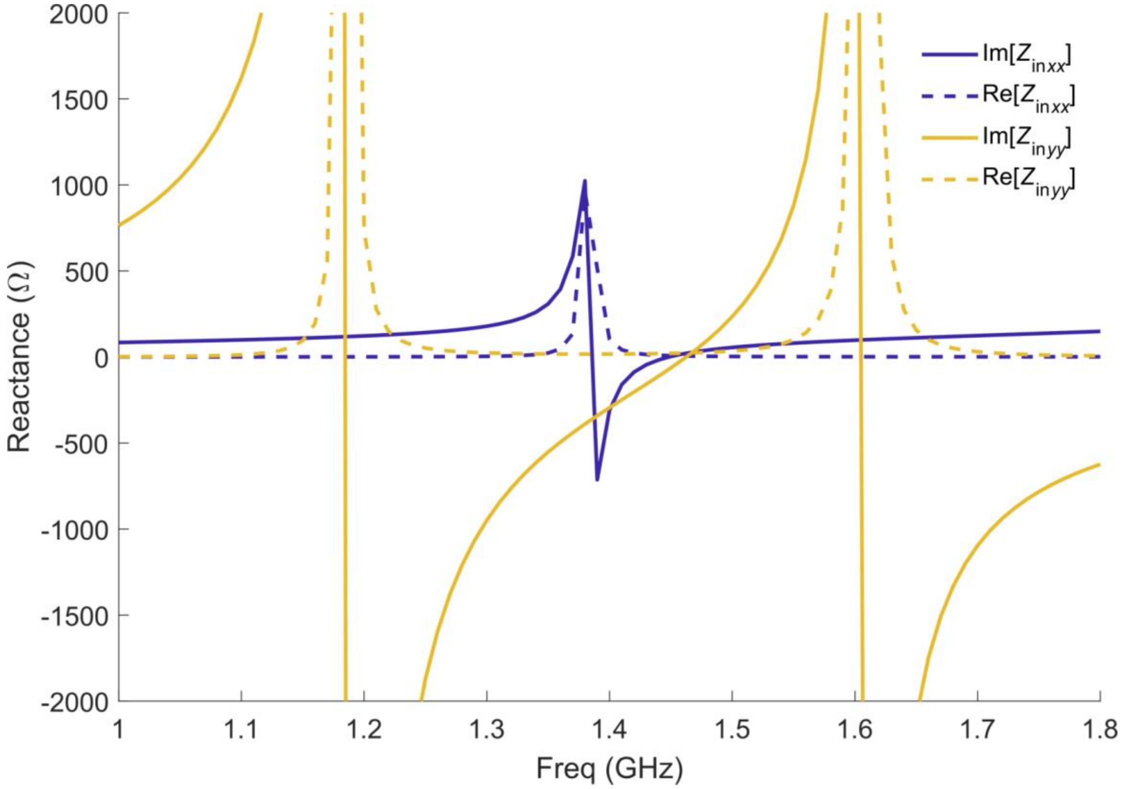

Supplementary Figure 11. Anisotropic effective input impedance of metasurface unit cell in a periodic environment, placed 33.6 mm above a ground plane.

When the metasurface unit cell is placed 33.6 mm above a ground plane (see Supplementary Fig. 9) the effective anisotropic input impedance can be calculated from the reflection coefficient. This input impedance is anisotropic due to the anisotropy of the metasurface. Note that the magnitude of the *y*-component of Z_{in} is much larger than the *x*-component near the L1 and L2 frequency bands, resulting in an approximately hard boundary condition ($|\mathrm{Im}\{Z_{in,xx}\}| << (|\mathrm{Im}\{Z_{in,yy}\}|)$.

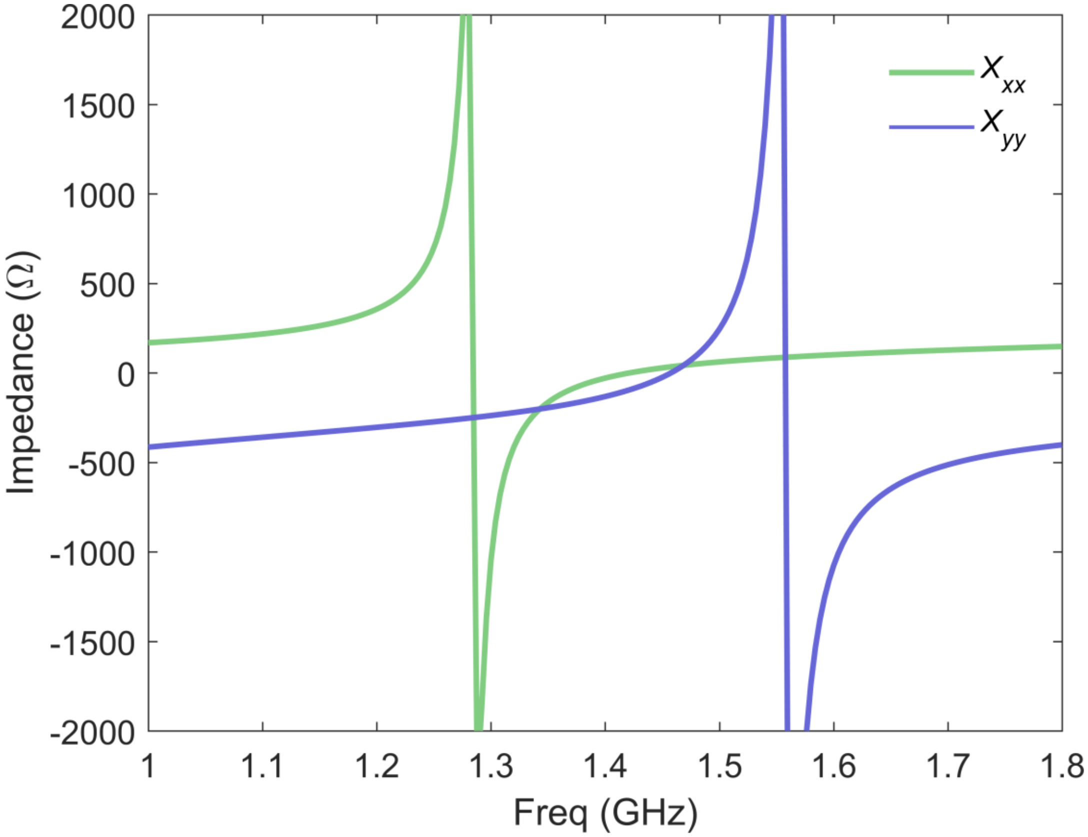

Supplementary Figure 12. Theoretical anisotropic metasurface reactance values required for optimized multi-band A-SBFA.

By increasing the parallel inductance of the unit cell we can shift the X_{xx} resonance to a lower frequency. Similarly, we can increase the series capacitance to decrease the magnitude of X_{yy} to fulfill the impedance surface requirements of the optimized A-SBFA design.

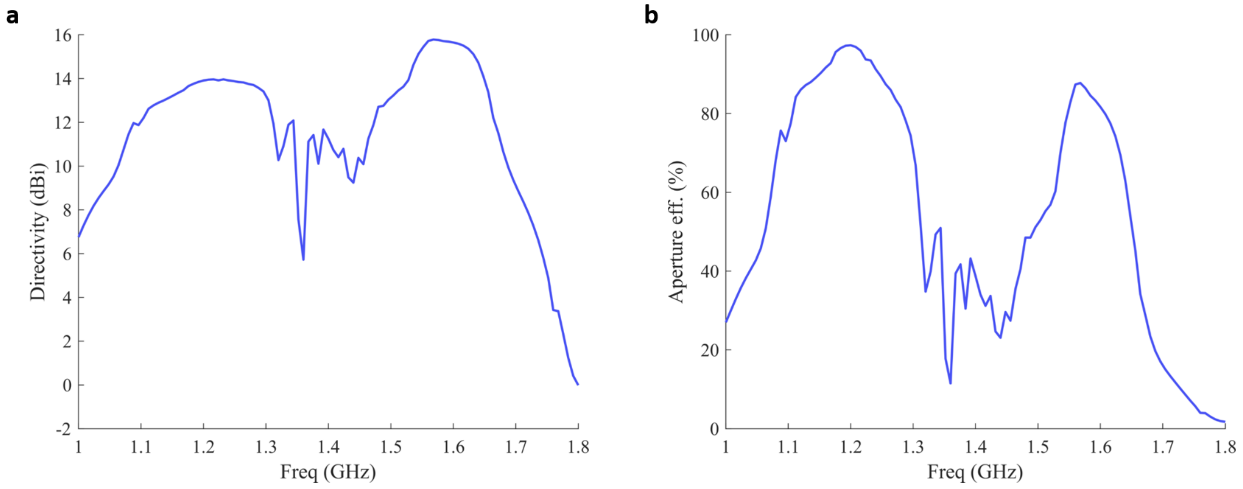

Supplementary Figure 13. Directivity and aperture efficiency of optimized multi-band A-SBFA.
Using new surface impedance parameters (Supplementary Fig. 12) the simulated directivity (**a.**) and aperture efficiency (**b.**) at L5 (1.176 GHz) are much improved. Simulated aperture efficiency is 95.6% at L5, 93.5% at L2, and 86.5% at L1.

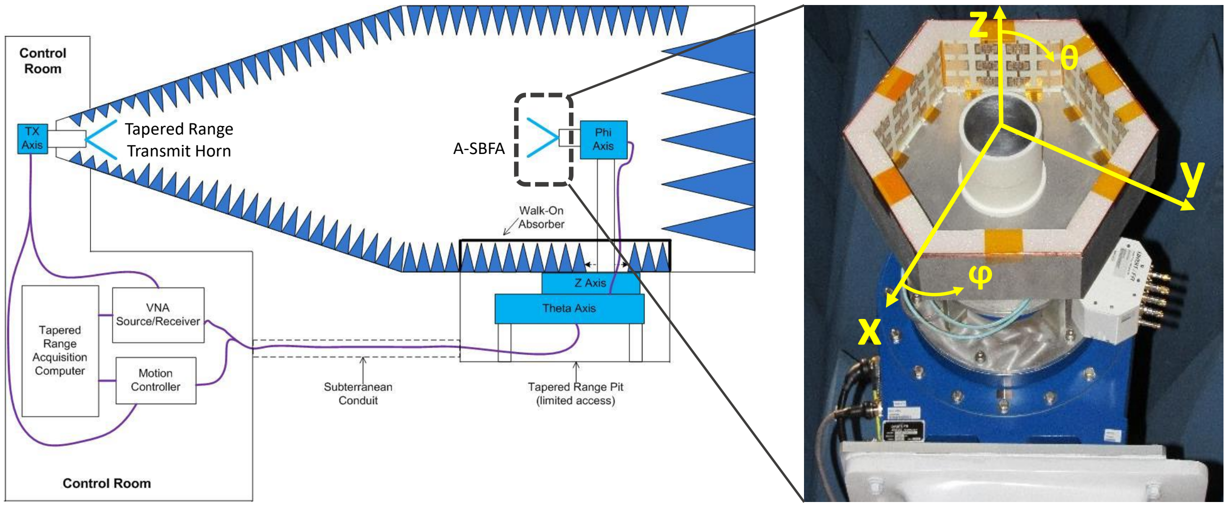

Supplementary Figure 14. Tapered anechoic chamber measurement set-up, with A-SBFA mounted on the 2-axis positioner. Far field radiation patterns of the A-SBFA were measured in a tapered anechoic chamber, shown in the above diagram. The 2-axis positioner was used to rotate the antenna, and enabled pattern measurements from 0° to 90° in the θ direction and from -180° to 180° in the ϕ direction.

Supplementary Tables

Supplementary Table 1. Sizes of SBFA dimensions in Supplementary Figure 1, given in terms of the wavelength at 1.4 GHz.

Dimension	Size (λ)
a	0.1551
b	0.0314
c	0.0097
d	0.0593
e	0.0994
f	0.2058
g	0.4777
h	0.4144
i	0.0962

Supplementary Table 2. Sizes of SBFA dimensions in Supplementary Figure 3, given in terms of the wavelength at 1.4 GHz.

Dimension	Size (λ)
a	0.1422
b	0.0314
c	0.0095
d	0.0711
e	0.1070
f	0.2151
g	0.4556
h	0.4133
i	0.0879
j	0.4827
k	0.8890

Supplementary Table 3. Sizes of metasurface dimensions in Supplementary Figure 8, given in terms of the wavelength at 1.4 GHz.

Dimension	Size (λ)
h_1	0.0303
h_2	0.1104
h_3	0.0133
h_4	0.0456
h_5	0.0286
h_6	0.0657
w_1	0.0236
w_2	0.0400
w_3	0.0221
w_4	0.0108
w_5	0.0641
w_6	0.0243
w_7	0.0195
w_8	0.0959
t_1	0.0011
t_2	0.0028
t_3	0.0024
t_4	0.0033
t_5	0.0028

Supplementary Notes

Supplementary Note 1

The metasurface lining the walls of the A-SBFA consisted of a periodic copper pattern etched on a thin dielectric substrate with a thickness of t_{s} and relative permittivity ϵ_{rs}. This substrate was separated from the aluminum walls of the cavity by a foam layer of thickness t_{f} and relative permittivity ϵ_{rf}. In order to determine the effective surface impedance, Z_{s}, of the metasurface, an equivalent transmission line circuit model was developed (Supplementary Fig. 9). In this model the metasurface is assumed to be infinitely periodic, and the aluminum walls of the cavity are approximated by a perfect electric conductor (*PEC*). Using well known transmission line equations, the input impedance at the foam/substrate interface can be calculated as

$$Z_{\mathrm{fin}} = jZ_{\mathrm{f0}} \tan(k_{\mathrm{f}} t_{\mathrm{f}}), \tag{1}$$

where Z_{f0} is the wave impedance of the foam, k_f is the propagation constant of the foam layer, and $j = \sqrt{-1}$. The input impedance of the dielectric/air interface, neglecting the surface impedance, can then be calculated by

$$Z_{\mathrm{sin}} = \frac{Z_{\mathrm{s0}}(Z_{\mathrm{fin}} + Z_{\mathrm{s0}} \tanh(jk_{\mathrm{s}} t_{\mathrm{s}}))}{Z_{\mathrm{s0}} + Z_{\mathrm{fin}} \tanh(jk_{\mathrm{s}} t_{\mathrm{s}})}, \tag{2}$$

where Z_{s0} and k_{s} are the wave impedance and propagation constant within the substrate, respectively. The input impedance of the entire system, Z_{in}, can be represented by an equivalent circuit where Z_{sin} and Z_{s} are connected in parallel. To determine Z_{s}, the reflection coefficient of the entire system must be determined, which can easily be found using a full-wave computational electromagnetics solver such as HFSS. For an anisotropic surface impedance Z_{s}, the reflection coefficients of *x*- and *y*-polarized waves, Γ_x and Γ_y, may be used to calculate the various components of the tensor

$$Z_{sx} = R_{xx} + jX_{xx} = \frac{Z_{\sin} Z_0 (\Gamma_x + 1)}{Z_{\sin} - Z_0 - \Gamma_x (Z_{\sin} + Z_0)} \quad (3)$$

$$Z_{sy} = R_{yy} + jX_{yy} = \frac{Z_{\sin} Z_0 \left(\Gamma_y + 1\right)}{Z_{\sin} - Z_0 - \Gamma_y (Z_{\sin} + Z_0)} \quad (4)$$

where Z_0 is the characteristic impedance of free space and R_{xx}, X_{xx}, R_{yy}, and X_{yy} are the real and imaginary parts of the diagonal tensor elements.
